# A Comparative Analysis Shows Morphofunctional Differences between the Rat and Mouse Melanin-Concentrating Hormone Systems

**DOI:** 10.1371/journal.pone.0015471

**Published:** 2010-11-17

**Authors:** Sophie Croizier, Gabrielle Franchi-Bernard, Claude Colard, Fabrice Poncet, Annie La Roche, Pierre-Yves Risold

**Affiliations:** Faculté de Médecine et de Pharmacie, Université de Franche-Comté, Besançon, France; Université Pierre et Marie Curie, France

## Abstract

Sub-populations of neurons producing melanin-concentrating hormone (MCH) are characterized by distinct projection patterns, birthdates and CART/NK3 expression in rat. Evidence for such sub-populations has not been reported in other species. However, given that genetically engineered mouse lines are now commonly used as experimental models, a better characterization of the anatomy and morphofunctionnal organization of MCH system in this species is then necessary. Combining multiple immunohistochemistry experiments with *in situ* hybridization, tract tracing or BrdU injections, evidence supporting the hypothesis that rat and mouse MCH systems are not identical was obtained: sub-populations of MCH neurons also exist in mouse, but their relative abundance is different. Furthermore, divergences in the distribution of MCH axons were observed, in particular in the ventromedial hypothalamus. These differences suggest that rat and mouse MCH neurons are differentially involved in anatomical networks that control feeding and the sleep/wake cycle.

## Introduction

The melanin-concentrating hormone (MCH) acts as a neurotransmitter/neuromodulator in the mammalian brain and is involved in various responses related to energy homeostasis, reproduction, sleep/wake cycle and reward [1]–[6]. Neurons producing this peptide are situated in the caudal hypothalamus and adjacent *zona incerta*. They project throughout the brain, from the olfactory bulb to the spinal cord [7]. Anatomy of the MCH system has been detailed in *Ratus norvegicus* [7],[8]–[10]. However, with the development of genetically engineered models, many experimental works are now done in *Mus musculus* [1],[2],[11]. It is often assumed that rat and mouse MCH systems are alike, but no true comparative studies have been performed to date. In rat, at least two sub-populations of MCH neurons exist, characterized by their birthdates, chemical phenotypes (cocaine- and amphetamine- regulated transcript, CART and neurokinin-3, NK3 co-expression) and projection patterns [10],[12]–[14]. However, the fine anatomy of the mouse MCH system is not known and little information exists on the CART expression and existence of sub-populations in murine MCH neurons. Such information is needed to provide sound interpretations of experimental studies in mouse models.

Because the brain cytoarchitectonic parcellations of the rat or mouse are very close, a comparative distribution of MCH cell bodies and axons is possible. In this work, MCH distribution was carefully analyzed in the mouse telencephalon and hypothalamus, and compared to rat MCH neuron distribution. We only considered obvious, undisputable divergences that may reveal interspecific functional particularities. We also verified the existence of sub-populations in the mouse MCH system, combining retrograde tract tracing, BrdU and multiple immunohistochemical experiments. We observed that these interspecific differences correlated with the pace of MCH genesis.

## Results

**Figure 1:**
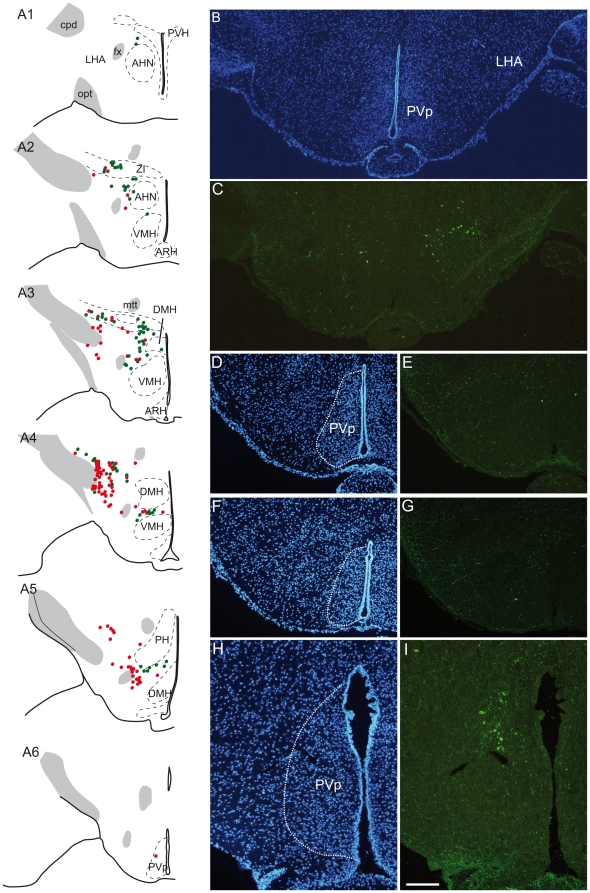
Distribution of MCH perikarya in the mouse hypothalamus. (A) Distribution of MCH/CART-positive and MCH-positive/CART-negative neurons, (respectively represented by green and red dots) on a series of line drawings of coronal sections through the mouse hypothalamus and arranged from rostral to caudal (A1 to A6). (B–G) Photomicrographs to illustrate the distribution of caudal-most MCH perikarya labeled by the sMCH-AS (C, E, G) at the level of the posterior periventricular nucleus (PVp). Sections were counterstained with DAPI to precisely identify cytoarchitectonic borders of the nucleus (B, D, F). Very few MCH perikarya are seen within this nucleus. (H–I) By contrast in rat, a group of MCH perikarya is labeled within the borders of the PVp. These neurons form a clear cell condensation. AHN: anterior hypothalamic nucleus, ARH: arcuate nucleus hypothalamus, cpd: cerebral peduncle, DMH: dorsomedial nucleus hypothalamus, fx: fornix, LHA: lateral hypothalamic area, mtt: mammillothalamic tract, opt: optic tract, PH: posterior hypothalamic nucleus, PVH: paraventricular nucleus hypothalamus, PVp: posterior periventricular nucleus hypothalamus, VMH: ventromedial nucleus hypothalamus, V3: third ventricle, ZI: *zona incerta*.

MCH cell body and MCH fiber distributions were analyzed by *in situ* hybridization and immunohistochemistry using three antisera (AS) raised against salmon MCH (sMCH), rat MCH (rMCH) and NEI. A DAPI counterstain or a Nissl stain of adjacent sections was also performed for cytoarchitectonic purposes. MCH perikarya were plotted on line drawings made from counterstained or Nissl stained sections (Figure 1A).

MCH projections were analyzed on rat and mouse coronal sections of the telencephalon and hypothalamus. Structures that were obviously and unambiguously differentially innervated by MCH axons were identified and observations verified on at least four brains in each species, using alternatively the sMCH-, rMCH- and NEI-AS. Obvious and unambiguous differences signify that structures were innervated in one species but not in the other with each of the three antibodies. More subjective differences were not considered since they were not supported by an adequate quantitative analysis.

### Distribution of MCH perikarya in mice and rats

On coronal sections, the rostral-most cell bodies were observed at the level of the caudal paraventricular nucleus (PVH), around and within the borders of the posterior part of the anterior hypothalamic nucleus using *in situ* hybridization or immunohistochemistry (Figure 1A). Caudal to PVH levels, corresponding to the tuberal hypothalamus, neurons were numerous close to the cerebral peduncle and in the perifornical region of the lateral hypothalamic area (LHA) as well as in the adjacent *zona incerta*. A few were seen in the dorsomedial capsule of the ventromedial nucleus. This general pattern of distribution is very similar to the MCH pattern described in rat [7],[15]. However, an interesting difference between the rat and mouse distribution was obvious at premamillary levels: while a dense group of MCH cell bodies is seen within the borders of the rat posterior periventricular nucleus (PVp), just ventral to the dorsal tuberomamillar nucleus [15], a similar group of neurons within the borders of the same nucleus could not be found in mouse. One or two perikarya could be seen in this nucleus, but they did not form a cell condensation as described in rat (Figure 1B-I) [see Figure 1 Atlas levels 32 and 33 of Hahn [15]].

In addition, perikarya in the olfactory tubercle like the ones described by Bittencourt et al. [7] in the rat, were not found in the mouse material (not illustrated).

### Hypothalamic MCH, CART and NK3 co-expressions and origin of cortical projections

**Figure 2:**
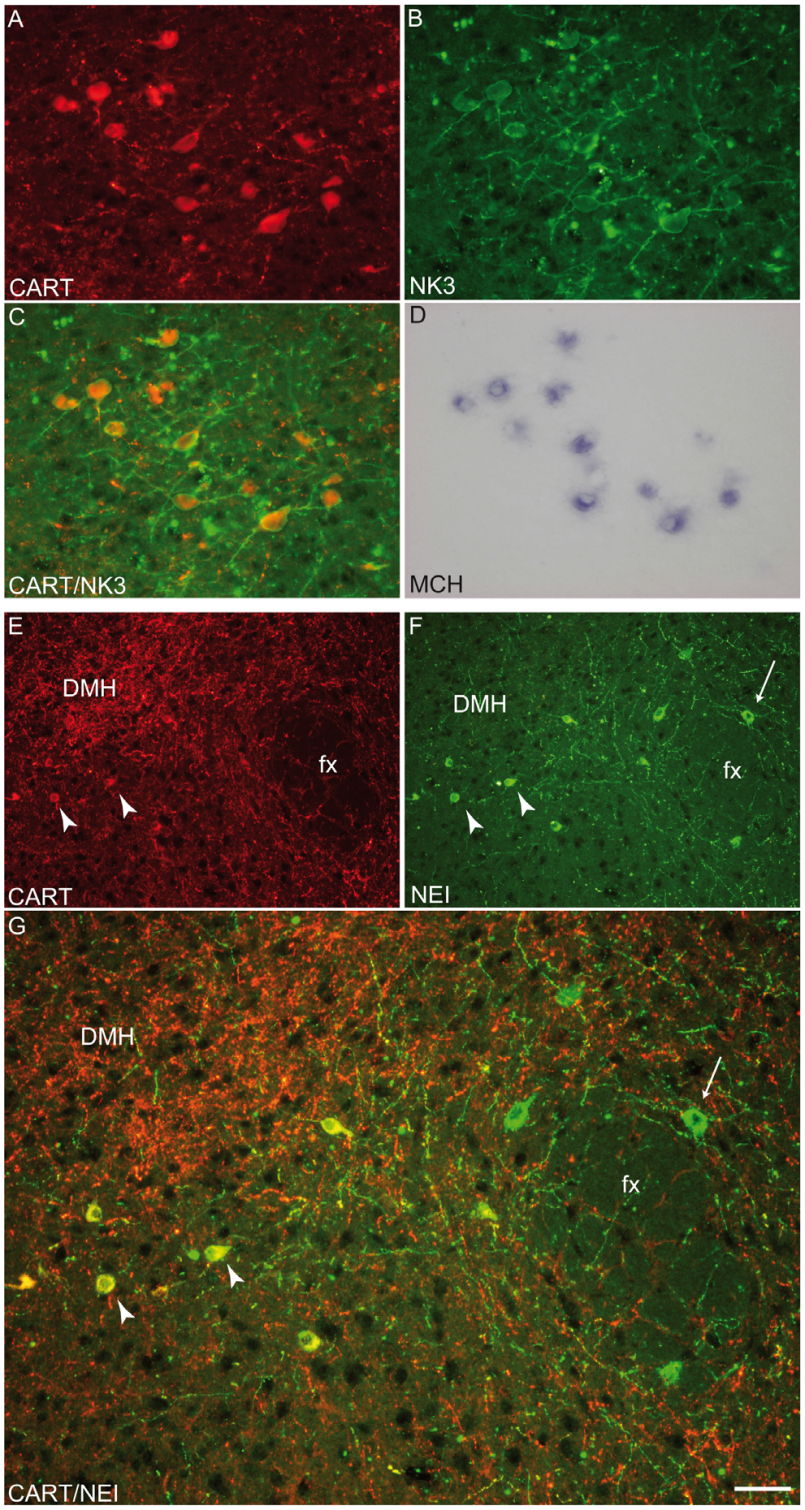
Co-expression of MCH/NEI, CART and NK3. (A–D) Photomicrographs to illustrate the co-expression of NK3 receptor and CART peptide by immunohistochemistry as well as the preproMCH (ppMCH) mRNA using *in situ* hybridization. CART and NK3 were first detected by immunofluorescence (A and B respectively, C). Then, the presence of the ppMCH mRNA in the same cell bodies was verified by *in situ* hybridization (D). All MCH positive neurons that expressed CART also contained NK3. (E–G) Photomicrographs showing NEI/CART (arrowheads) or NEI (arrows) labeled neurons. Most NEI-positive neurons localized medially to the fornix expressed CART. NEI and CART co-expression was less frequent lateral to the fornix (G). DMH: dorsomedial nucleus hypothalamus, fx: fornix.

CART is expressed in 66% of all hypothalamic MCH cell bodies in rat hypothalamus [10]. These neurons also express the NK3 receptor [13]. Using a dual immunohistochemical approach on cryostat cut sections, perikarya labeled by the sMCH- or NEI-AS were also labeled by CART monoclonal antibodies (Figure 2E–G). These neurons represented 44.5% of the whole population as compared to 66% in rat (Table 1). By detecting CART, NK3 (immunohistochemistry) and MCH (*in situ* hybridization) on the same sections, all MCH/CART neurons were found to also express NK3, as in rat (Figure 2A–D). No other CART or NK3 neurons could be observed in the caudal LHA. Most of the rostral MCH neurons in the *zona incerta* or medial to the fornix expressed CART/NK3, but most of the lateral and caudal MCH neurons did not. A similar segregation of the two sub-populations was reported in the rat [10].

**Table 1:** CART expression in MCH neurons.

Mean of number of MCH or NEI cell bodies	% MCH- or NEI-CART/MCH or NEI
706±110	44,49±5,05

Proportion of MCH neurons containing CART in the diencephalon of five mice. Between one and three series were counted for each mouse. Mean±SD.

**Figure 3:**
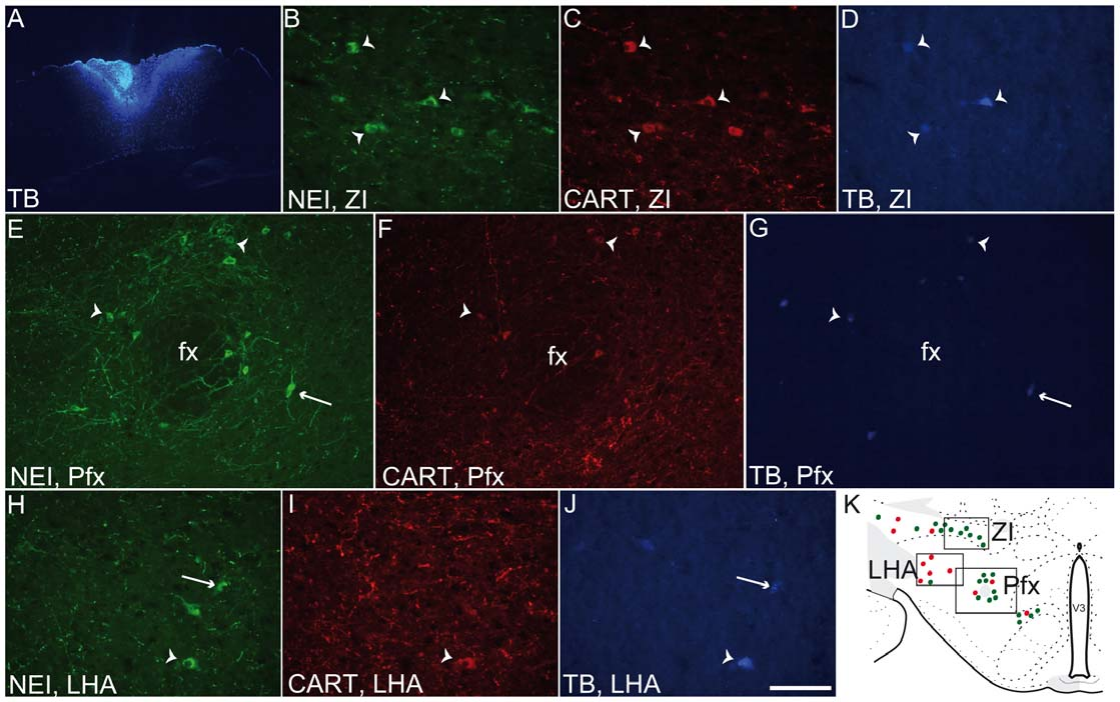
Origin of MCH projections in the mouse cortex. (A) Photomicrograph illustrating a true blue (TB) injection site in the mouse medial cerebral cortex. (B–J) Photomicrographs showing NEI/CART labeled neurons containing true blue (arrowheads) or neurons containing both NEI and true blue but not CART (arrows). Triple labeled cells were abundant in the *zona incerta* (ZI) and medial perifornical regions (Pfx), while double labeled cells were mostly found in the lateral hypothalamic area (LHA). The schem in K shows the localization of the three regions and a schematic distribution of NEI/CART and NEI positive/CART negative neurons represented by green and red dots respectively. fx: fornix, V3: third ventricle.

80% of cortically projecting MCH neurons expressed CART in rat [13]. A retrograde tract tracing experiment was conducted to verify that MCH/CART neurons project in the cerebral cortex in mouse as well. True blue was injected in the dorso-medial cortex (dorsal cingulate/motor fields) of three mice (Figure 3). As expected, many retrogradely labeled neurons were found in the lateral hypothalamus and *zona incerta*. Using dual immunohistochemistry staining on these sections, we found that 60% of retrogradely labeled MCH neurons expressed CART (Table 2). Then, the MCH/CART sub-population takes a larger part of the whole of MCH cortical projections: these neurons represent only 44.5% of the population but 60% of cortically projecting MCH cells. However, the MCH-only cortical projection is not marginal and the balance of MCH (40%) vs MCH/CART (60%) cortical inputs is more equilibrated in mouse than in rat (20% and 80% respectively). Observation of MCH and CART expression in cortical axons corroborated this result (not illustrated). MCH axons not labeled by the CART antibodies were abundant, while in rat these axons are rare. Furthermore, many CART axons not labeled by MCH- or NEI-AS were observed in mouse. In rat cerebral cortex, CART-positive/MCH-negative axons were very rare [see 10], suggesting an additional origin for the innervations by CART axons in mouse cerebral cortex.

**Table 2:** MCH cells projecting in the mouse cerebral cortex.

Mice	Mean of MCH-TB neurons±SD	% MCH+TB neurons which were CART-positive±SD
#31	73,33±8,33	60,4±7,2
#32	101±28,28	61,5±13,58
#33	48,67±10,26	60,7±11,66
% total	-	60,9

Number of MCH neurons retrogradely labeled after true blue injection in the cerebral cortex and, among them, percentage of MCH/CART-positive neurons retrogradely labeled. Two series of section were counted in case 32, and three in cases 31 and 33.

In rat, most spinally projecting MCH cells do not contain CART [10]. Similarly in mouse, double labeled MCH/CART axons in sections through the spinal cord were very rare, suggesting that, as in rat, spinal projections arise mostly from MCH perikarya that do not express CART (not illustrated in mouse), (see 10 for the rat data).

### MCH genesis

**Figure 4:**
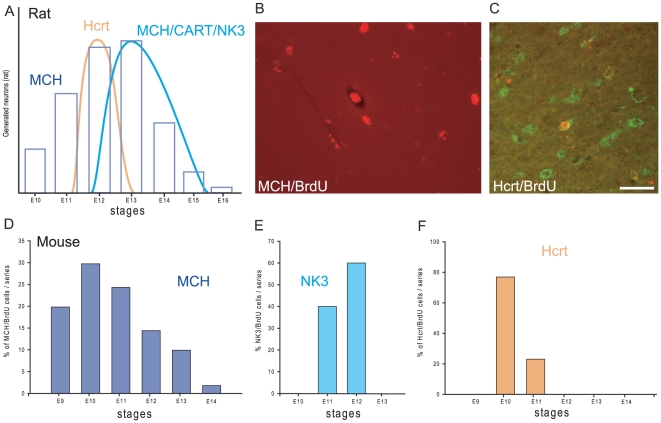
MCH neuron genesis in the mouse hypothalamus. (A) Histogram summarizing precedent findings in the rat concerning the genesis of MCH, MCH/CART/NK3 and Hcrt neurons in the hypothalamus [12],[18]. (B–F) A similar study combining BrdU, immunohistochemistry and *in situ* hybridization was conducted in mouse to compare the birthdates of MCH and Hcrt neurons. (B) Photomicrograph illustrating a neuron double-labeled for BrdU (red immunohistochemistry) and MCH (*in situ* hybridization). (C) Dual immunofluorescence to identify Hcrt (green) and BrdU in the same neurons. Only neurons displaying a densely BrdU-labeled nucleus were taken into consideration. (D–F) Histograms of MCH/BrdU, NK3/BrdU and Hcrt/BrdU neurons. MCH/BrdU positive neurons are seen from E9 to E14, with a peak at E10 (D). NK3/BrdU positive cells in the *zona incerta*/LHA were observed only at E11 and E12 (E). On the same material, double labeled Hcrt/BrdU perikarya were observed mainly at E10 (F). LHA: lateral hypothalamic area.

Past data obtained in rat showed that the whole MCH population is generated over several gestational stages (from embryonic day 10 (E10) to E16, with a peak at E12/E13) (Figure 4A) [12]. This pattern of MCH genesis was compared to the production of the hypocretin/orexin (Hcrt)-containing neurons; these cells co-localize with MCH neurons, project with a similar pattern throughout the brain and are involved in similar functions [6],[16],[17]. In contrast to MCH, these cells are generated in one sharp peak at E12 in the rat [18]. With regard to MCH sub-populations, it seems that MCH, Hcrt and MCH/CART cell bodies are generated in successive waves: the first wave gives rise to spinally projecting MCH neurons at E11, then Hcrt neurons are generated at E12, and MCH/CART neurons are produced slightly later at E12/E13 [12],[14],[18].

A similar BrdU experiment was done in mice to verify if MCH, Hcrt and MCH/CART neurons were generated through a similar pattern. Double labeled MCH/BrdU perikarya were observed in E9 to E14 BrdU injected embryonic sections, but their production peaks at E10 (Figure 4B–D). The distribution of these neurons broadly followed a lateral-to-medial gradient, as already described in rat [12],[19]–[21]; the overwhelming majority of neurons born before or on E10 were located lateral to an arbitrary vertical line passing through the fornix on coronal sections, while most of those generated later were located medially in the hypothalamus and *zona incerta*. NK3/BrdU double labeled neurons were counted in the tuberal LHA; these cells were generated between E11 and E12, indicating that MCH/CART/NK3 co-expressing neurons are generated later than most MCH only neurons (Figure 4E).

On the same mouse material, close to 80% of all the double labeled Hcrt/BrdU neurons were observed in the E10 material with only a few in E11 animals (Figure 4F).

### Differences in the distribution of MCH axons in mouse and rat

In mouse, MCH axons are observed throughout the brain, from the olfactory bulb to the spinal cord, recalling MCH distribution in many species including rat [7]. However, three very clear differences were found compared to the latter species.

**Figure 5:**
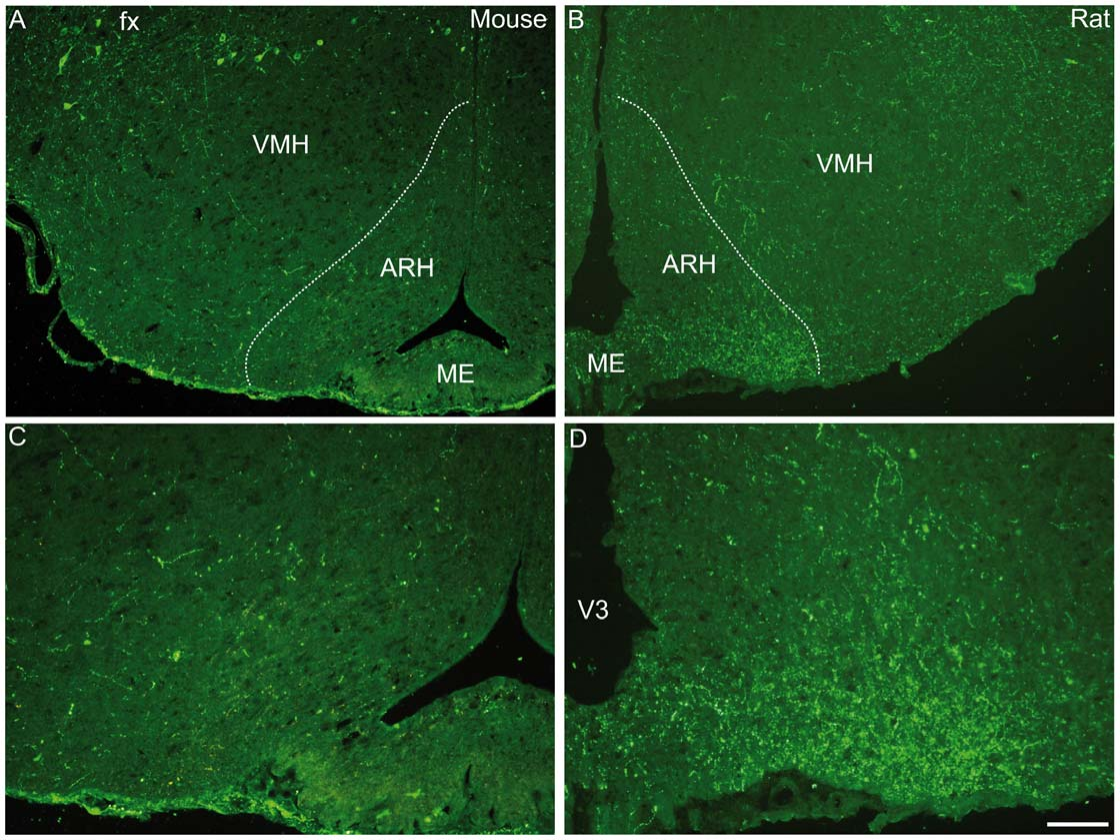
MCH axons in the arcuate nucleus. Photomicrographs illustrating the MCH labeling in the arcuate nucleus (ARH) in mouse (A and B) or rat (C and D) hypothalamus. In mouse, few MCH fibers were observed in the ARH whereas a dense MCH inputs were present in a ventral part of the rat ARH.

**Figure 6:**
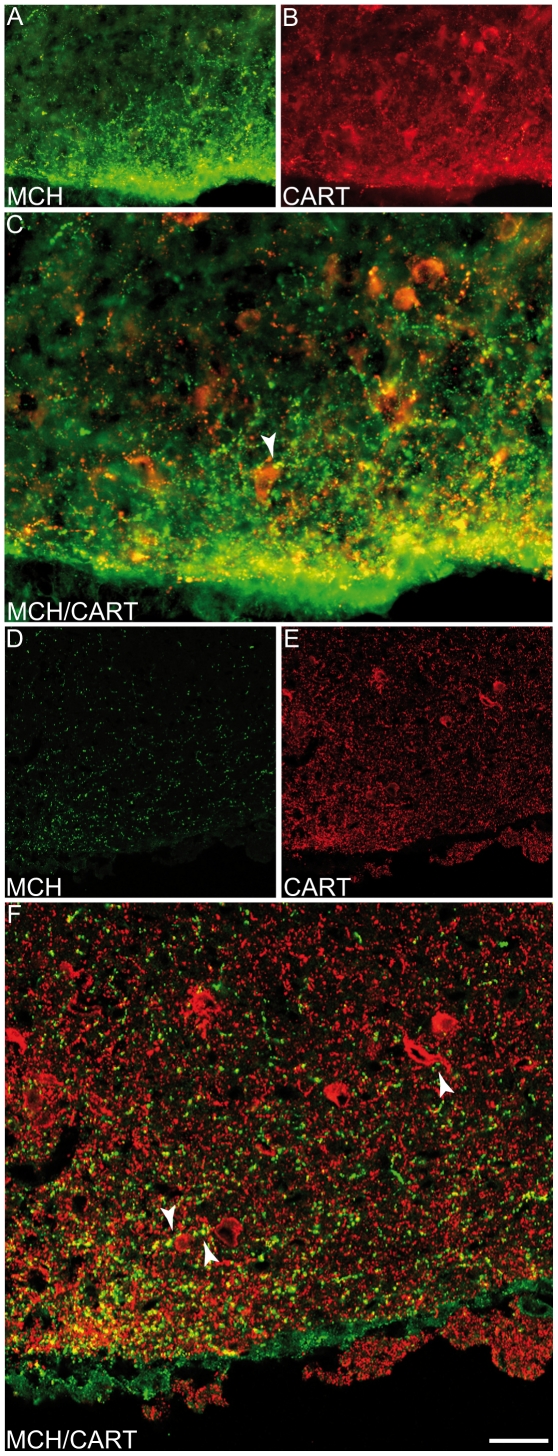
Innervation of POMC neurons by MCH axons. Standard immunofluorescence (A–C) or confocal microscopy (D–F) to illustrate double labeling for CART and MCH in the rat ventral ARH. MCH axons appeared to make synaptic contacts on POMC cells that were labeled by the CART-AS.

The most obvious and striking difference concerned the hypothalamus. In the rat, abundant innervations of the ventral part of the arcuate nucleus is found (Figures 5 and 6) [7]. Using dual immunocytochemistry and confocal microscopy, MCH axons appeared to synapse on proopimelanocortin (POMC) cells that were labeled by the CART-AS. Numerous MCH axons were also seen in the adjacent median eminence and pituitary stalk. On our mouse material, a dense terminal field in the arcuate nucleus was never found and the ventromedial hypothalamus as a whole contained only a few axons (Figure 5).

**Figure 7:**
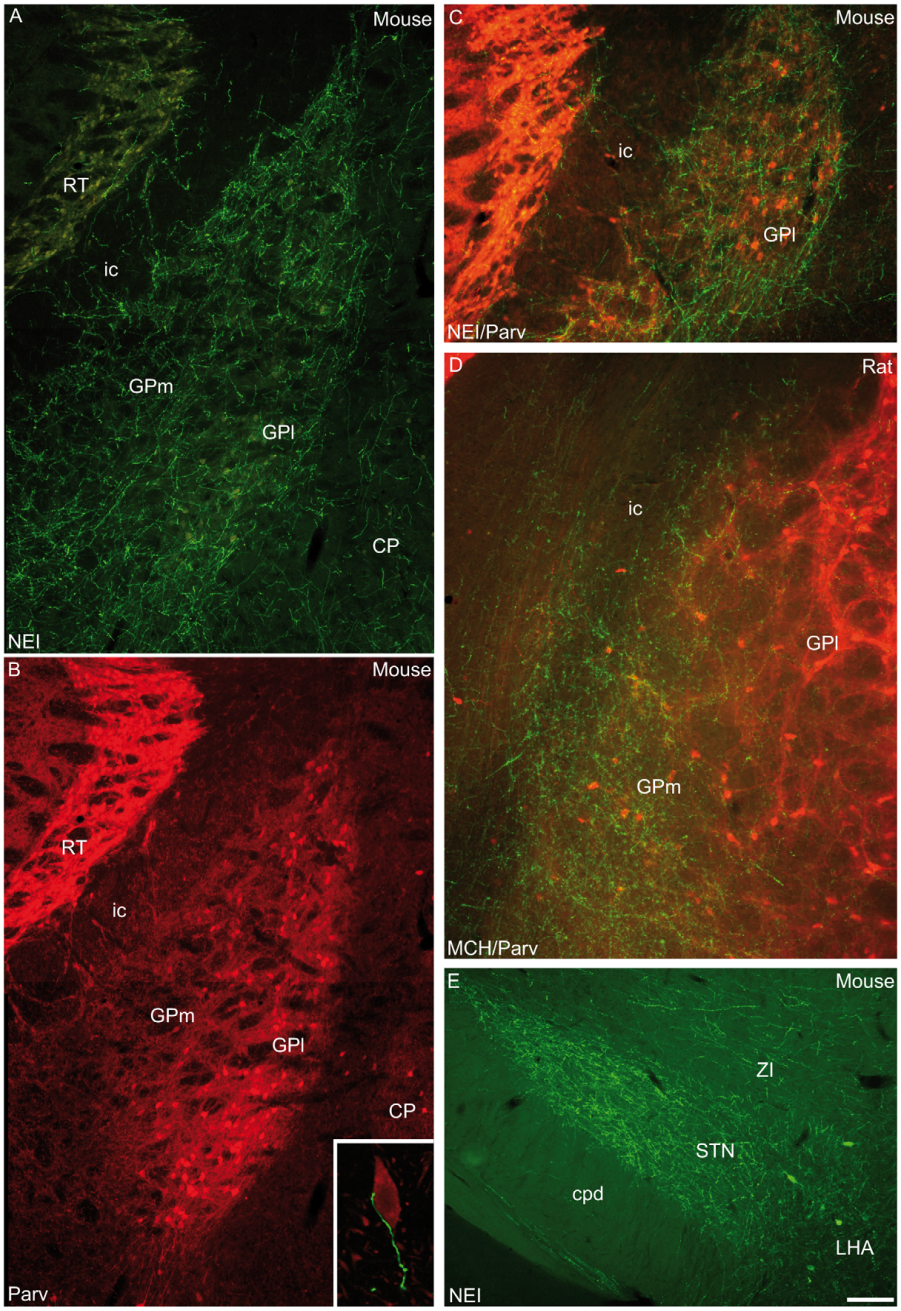
MCH axons in the Globus Pallidus. (A–C) Photomicrographs to illustrate the MCH innervation of the mouse globus pallidus (GP). MCH axons labeled by the NEI-AS (A) or sMCH-AS (C, green labeling) are observed throughout the nucleus. Medial (GPm) and lateral (GPl) parts are innervated. GPm contains few parvalbumine (Parv)-containing cell bodies, while GPl contains many such neurons (B, red labeling in C). MCH axons are seen close to Parv neurons in the GPl: insert in B is a confocal image showing a MCH axon (green) innervating a Parv perikarya (red). (D) Double labeling for MCH/Parv in rat globus pallidus. Only medial regions of the GP were innervated by MCH fibers, while lateral parts of the nucleus do not contain a MCH innervation. (E) Photomicrograph showing intense NEI innervations of the mouse subthalamic nucleus (STN).

In the pallidum, both medial and lateral parts of the globus pallidus contained a dense MCH innervation in the mouse brain (Figure 7). The lateral part is characterized by the presence of parvalbumin-containing cell bodies in contrast to the medial globus pallidus, which is poor in parvalbumin-expressing cells [22] . Using a double immunocytochemical approach, MCH axons were observed in the immediate vicinity of parvalbumin cells, suggesting axosomatic synaptic contacts (Figure 7A–C). On the rat material, although both pallidal compartments exist, only the very medial globus pallidus contained a dense MCH input, while lateral parts of the nucleus contained only sparse passing MCH axons (Figure 7D). The subthalamic nucleus, which is anatomically related to the globus pallidus, was intensely innervated by MCH axons in both rat and mouse (Figure 7E).

**Figure 8:**
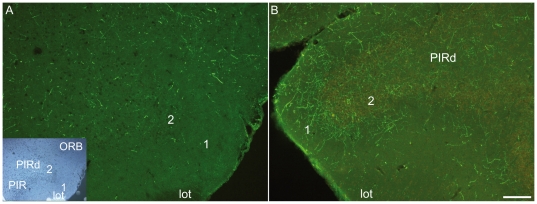
MCH axons in the piriform cortex. Photomicrographs illustrating the MCH innervation of the piriform cortex (PIR) in mouse (A) and in rat (B). Insert in A is a darkfield wiev of the same section for cytoarchitectonic purposes. The indentation of this cortical field just dorsal to the lateral olfactory tract (lot) is very clear. In rat, many MCH fibers are observed in layers 1 and 2 of the dorsal piriform area, whereas very few are seen in mouse. PIR: piriform cortical field, PIRd: dorsal region of the piriform area, ORB: orbital area.

A very abundant MCH projection is especially dense in the dorsal region of the piriform cortex, just dorsal to the lateral olfactory tract. In this region, layer 2 makes an indentation on coronal sections and is thus easy to recognize. In the mouse, a region of the piriform cortex with similar characteristics was identified, but did not contain a MCH terminal field (Figure 8).

## Discussion

In both rat and mouse, many MCH cell bodies are observed in the lateral hypothalamic area. These neurons send their axons throughout the brain from the cerebral cortex to the spinal cord [7],[15]. In rat, two MCH sub-populations were described based on CART and NK3 expressions, projection patterns and birthdates [10],[12]–[14],[23]. In mouse, we find again evidence for the existence of two similar sub-populations: MCH/CART/NK3 cells bodies were observed and these neurons are generated after MCH-only cell bodies (E12 vs E10). However, clear differences were also noticed. In rats MCH/CART/NK3 neurons represent 2/3 of the whole MCH population [12], while in mouse they made up only 44.5% of all MCH neurons. In this latter species, 60% (against 80% in rat; [13]) of MCH cortical projections arise from MCH/CART neurons. Other differences between the rat and mouse MCH systems concerned specific projections in the piriform cortex, globus pallidus and ventromedial hypothalamus.

These anatomical inter-specific particularities indicate that MCH neurons are involved in distinct telencephalic and hypothalamic networks, which may reflect important functional differences. For example, regardless of the two cell types, we observed in mice a significant innervation of the external part of the globus pallidus and particularly of the lateral pallidal parvalbumin-containing neurons. A similar projection does not exist in rat [7]. Pallidal parvalbumin neurons receive a strong enkephalinerigic striatal input and project to the entopeduncular nucleus and substantia nigra as well as the subthalamic nucleus also innervated by MCH axons in both rat and mouse [22]. These observations suggest that, in addition to its well described action in motivation through connections with ventral striatal circuits and particularly the accumbens nucleus (well described in the literature, [5],[24],[25]), MCH may also be involved in cognitive components of volitional motor behavior in mouse. This may be related to the important locomotor increase reported in MCH−/− and MCHR1−/− mice [1],[26],[27].

Given the strong literature support for a role for MCH in energy homeostasis [3],[28]–[30], the divergent innervations by MCH axons of the rat and mouse piriform cortex, and, more obviously, of the hypothalamic ventral arcuate nucleus, is important to consider. This part of the arcuate nucleus is rich in POMC/CART neurons that play a key role in feeding [31]–[33]. This region, including POMC neurons, is intensely innervated by MCH axons in the rat. The latter results suggest a direct control of POMC neurons that may in part account for the recognized orexigenic role of MCH in this species [34]. However, the absence of a clear terminal field in the mouse arcuate nucleus indicates that MCH may not participate in the same way in the control of POMC neurons in this species. It is very tempting to correlate the absence of MCH arcuate projections with the lack of MCH cell bodies in the PVp. In the rat, dense arcuate projections were never reported after anterograde tract tracing from the LHA [35], rostromedial *zona incerta* [36] and dorsomedial nucleus [37], where MCH neurons are located. Therefore, the PVp appears to be a good candidate because this nucleus is part of the periventricular zone of the hypothalamus, like the arcuate nucleus. Furthermore, the ventral premammillary region sends very dense projections to the arcuate nucleus [38]. Unfortunately, in absence of comparative experimental studies, it is very difficult to draw more definitive conclusions about the functional significance of these inter-specific differences [however see as well 33].

**Figure 9:**
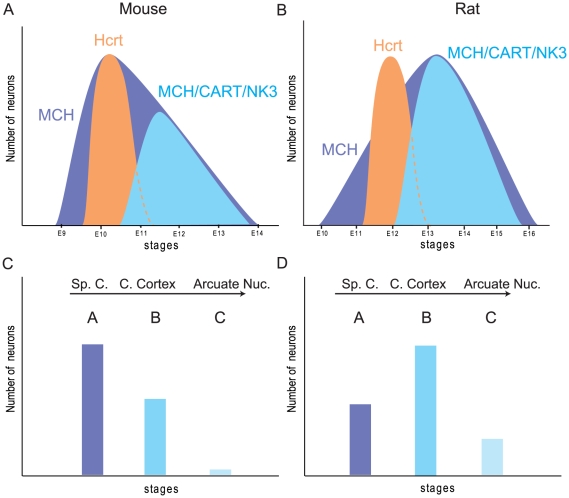
Diagrams summarizing differences between rat and mouse MCH systems. Summary diagrams to compare the genesis of MCH, Hcrt neuron populations and MCH/CART/NK3 sub-population. In mouse (A), both peaks of MCH and Hcrt production are at E10. MCH/CART/NK3 neurons are generated after E10 and constitute less than half of the whole MCH population. In rat (B), the peak of MCH production is late compared to the peak of Hcrt genesis, and in this species MCH/CART/NK3 neurons form 2/3 of the whole MCH population. It can be hypothesize that these different patterns of genesis have an impact on the organization of the MCH neuron system in the two species: in mouse (C), MCH type A neurons is preeminent compared to MCH type B neurons. On the contrary, MCH type B neurons is better represented in rat (D), and a group of lastly generated cells with a periventricular distribution and projections in the arcuate nucleus (MCH type C neurons) can be observed. This last group is lacking in mouse.

Without any doubt, dissimilarities between rat and mouse MCH systems are related to divergences in their developmental differentiation (Figure 9). In agreement with classical birthdating studies, we observed that neurons settle in the hypothalamus following a lateral to medial gradient in mouse as in rat; first-wave generated neurons lay close to the cerebral peduncle, and lastly produced cells are periventricular [19]–[21]. In rat, the peak of MCH genesis correlates with the production of MCH/CART/NK3 cell bodies, and is slightly late compared to the peak of Hcrt neuron genesis. In mice we observed that both MCH and Hcrt peaks coincided, but that MCH/CART/NK3 neuron production is still later than Hcrt genesis. We can conclude that compared to Hcrt genesis, the peak of MCH production is a little more precocious in mouse than in rat and MCH/CART neurons are less abundant. A slightly more precocious MCH production could also explain the absence of MCH neurons in the PVp. We know that these neurons are produced last in the rat [12]; maybe MCH neuron production in mice ends before the differentiation of a MCH cell group in this periventricular nucleus. In the past, we used a specific nomenclature in the rat to identify MCH neuron sub-populations [23]: MCH type A neurons for spinally MCH projecting cells, and MCH type B neurons for MCH/CART cortically projecting cells [13],[14],[23]. We also suspected that, in the rat, the latest produced cells (including those in the PVp), which are never labeled after retrograde injections in the cerebral cortex nor the spinal cord, could be grouped into a MCH type C sub-population [14]. In rat, MCH type B neurons are prominent. In mouse, MCH type A neurons are more abundant, and as their production peak is coincident with the Hcrt peak, they may also project with these neurons in the cerebral cortex. With an early peak of MCH genesis, fewer cells are observed medial to the fornix and MCH type C neurons are rare in this species, with very few MCH projections in the arcuate nucleus.

To conclude, the rat and mouse MCH systems are not identical. Differences exist in the distribution of cell bodies and fibers. Therefore, these neurons are involved through species specific mechanisms in the control of feeding behavior and sleep/wake cycle, and pace of genesis may explain these differences.

## Materials and Methods

### Animals

All animal use and care protocols were in accordance with institutional guidelines (all protocols were approved and investigators authorized). Long Evans rats (n = 4) were obtained from Charles River Laboratories, L'Arbresle, France, Swiss mices (n = 34) from Janvier, Le Genest-Saint-Isle, France.

### Comparative expression of sMCH/rMCH/NEI, CART and NK3

Adult male rats (n = 4) and mice (n = 10) were perfused as previously described [12] with NaCl 0.9% followed by ice-cold 1% or 4% paraformaldehyde (PFA) fixative in 0,1 M phosphate buffer. The brains were removed, post-fixed in the same fixative for several hours at 4°C, immersed overnight at 4°C in a 15% sucrose solution, and then quickly frozen. Brains of rats or mice perfused with PFA 1% were serially cut into 10 µm coronal sections on a cryostat-microtome, mounted on gelatinated slides and stored at −40°C until treatment. Those perfused with PFA 4% were cut in five (rat) and four (mouse) series of 30 µm coronal thick sections, collected in a cryoprotector solution (1∶1∶2 glycerol/ethylene glycol/phosphate buffered saline or PBS) and stored at −20°C.

### Immunohistochemistry

After rinsing in PBS+0.3% Triton X100, sections were incubated with the primary antiserum (AS), at the appropriate dilution, overnight or during 24 h at room temperature for cryostat sections and free-floating sections, respectively (see Table 3; [39]). The labeling was then revealed with secondary donkey anti-mouse IgG conjugated to Alexa Fluor-555 (1/800 or 1/1000, Invitrogen) or a goat anti-rabbit IgG conjugated Alexa Fluor-488 (1/800 or 1/1000, Invitrogen) for 1 h at room temperature. All antisera were diluted in PBS containing 0.3% Triton X100, 1% bovine serum albumine, 10% lactoproteins and 0.01% sodium azide except for monoclonal antibodies which were diluted only in a PBS-Triton solution [10].

**Table 3:** Antibodies used for immunohistochemistry.

Antibodies	Species	Antigen	Source	Dilution (cryostat sections)	Dilution (free-floating sections)
sMCH	Rabbit	Salmon MCH	EA3922 [39]	1/200	1/2000
rMCH	Rabbit	Rat MCH	EA3922 [39]	1/200	-
NEI	Rabbit	Rat NEI	EA3922 [39]	1/200	1/2000
CART	Mouse	Rat CART	Dr Clausen, Danemark	1/1000	1/4000
CART	Rabbit	CART (61–102)	Phoenix Pharmaceuticals, USA	1/3000	-
Parvalbumin	Mouse	Human parvalbumin	Swant, Suisse	1/5000	1/10000

The specificity of the salmon MCH-AS was verified by liquid phase inhibition, dot blot, and immuno-affinity [42],[39] as well as by immunohistochemistry/*in situ* hybridization double labeling [43],[44]. The monoclonal CART-AS was generously provided by Dr Clausen (see acknowledgements).

### *In situ* hybridization

The preproMCH (ppMCH) RNA probe was made in our laboratory [12]. The rat MCH cDNA was obtained by reverse transcription/polymerase chain reaction from total RNA of adult rat brain following a protocol described by Brischoux et al. [12]. The antisense (complementary to cellular mRNA) and control sense (identical to cellular mRNA) probes were produced by using the RNA transcription kit (Roche) and were digoxigenin (DIG)-UTP-labeled.

Standard *in situ* hybridization: frozen sections were post-fixed in 4% paraformaldehyde in 0,1 M phosphate buffer and digested with proteinase K (1 µg/ml, Roche) for 30 min at 37°C. Slides were incubated for 8 min in 0.1 M triethanolamine (TEA), pH 8.0, and then for 5 min at room temperature in 100 mL 0.1 M triethanolamine (TEA)+500 µL acetic anhydride followed by a decarboxylation in active diethyl pyrocarbonate (DEPC).

Sections were then rinsed briefly with 5× SSC (standard citrate sodium buffer) then incubated for 2 h in prehybridization buffer at 56°C. After rinsing in 0,2× SSC, the sections were incubated overnight at 56°C for MCH, in humid chambers, with 50 µl hybridization buffer containing 5% Denhardt's and 50 ng labeled RNA probes. After rinsing with 5× SSC, sections were incubated successively in 0,2× SSC at 56°C (1 h30) and 0,2× at room temperature (5 min). They were incubated in anti-DIG Fab fragments conjugated to alkaline phosphatase (1/1300, overnight) and revealed with enzyme substrate NBT-BCIP (overnight, at room temperature).

Control hybridization, including hybridization with sense DIG-labeled riboprobes was realized.

#### Co-detection of CART, NK3 or Parvalbumin (Parv) and MCH or NEI

A regular double indirect immunofluorescence with different fluorochroms was performed for co-detection of CART/sMCH or NEI, parvalbumine/sMCH or NEI. Sections were first incubated with the first primary antibody and revealed following the same conditions as described earlier, and then incubated with the second primary antibody and revealed with a secondary antibody (goat anti-rabbit IgG conjugated to Alexa Fluor-488 (1/800, Invitrogen) for MCH and NEI and with a secondary goat anti-rabbit IgG conjugated to Alexa Fluor-488 (1/800, Invitrogen) for CART and Parv). Double labeled MCH or NEI/CART neurons were counted on 1 to 3 series per brain of 5 brains.

A triple labeling was performed for the detection of NK3/CART/MCH, through the following procedures. CART and NK3 neurons were detected both by indirect immunofluorescence using, respectively, a secondary donkey anti-mouse IgG conjugated to Alexa Fluor-555 and a secondary goat anti-rabbit IgG conjugated to Alexa Fluor-488, then MCH was detected by *in situ* hybridization (see above).

### Tracer injections

Mouse adult male (40 g, n = 3) were anaesthetized with an intraperitoneal (IP) injection of a mixture of xylasine and ketamine (1 mg/100 g of body weight and 10 mg/100 g, respectively) and placed in a stereotaxic instrument. About 300 nL of a 4% true blue (Sigma) solution in distilled water was injected with a Exmire microsyringe (MS-N05, ITO corporation, Italy) into medial cerebral cortal fields over a period of 15 min. Four days after the true blue injections, animals received an intraperitoneal injection (IP) of 7% chloral hydrate and were perfused as described above.

### BrdU injections

Six pregnant Swiss mice (at stages E9 to E14) were given an IP injection of 5-bromo-2-deoxyuridine (BrdU; Sigma, France; 100 mg/kg body weight, dissolved in 0.07 M NaOH and warmed to 65°C, [40]). Young adult male offspring (n = 21) of the BrdU-injected females were anaesthetized with an IP of 7% chloral hydrate (1 ml per 200 g body weight, Prolabo). They were then perfused with 1% PFA as described above.

For the combined detection of BrdU and MCH, Hcrt or NK3, an acid hydrolysis was first performed. Double immunofluorescence staining was then done as previously described [12]. This study was accomplished by combining BrdU immunohistochemistry and MCH *in situ* hybridization or Hcrt immunohistochemistry. The *in situ* hybridization was done first as described followed by the BrdU detection. Double labeled MCH/BrdU neurons were counted on 6 to 9 series per brain of 2 or 3 brains per stage, double labeled Hcrt/BrdU neurons were counted on 5 to 11 series per brain of 2 to 4 brains per stage and double labeled NK3/BrdU neurons were counted on 2 to 5 series per brain of 1 to 3 brains per stage.

### Cytoarchitecture and nomenclature

Sections were analyzed on an Olympus fluorescence microscope BX51 or an Olympus confocal microscope Fluoview FV1000 BX configuration. Images were obtained through a DP50 or DP75 numeric camera (Olympus, France), using the analySIS software or Fluoview FV1000 (Olympus, France).

Anatomical structures were identified according to Swanson [41]. Hahn [15] provided the most detailed description of the rat MCH perikarya distribution in the hypothalamus yet. Bittencourt et al. [7] provided the best description of MCH projections. We are making frequent references to these works. We choose to use a matching hypothalamic divisions and nomenclature in both rat and mouse. A careful examination of Nissl stained sections of mouse hypothalami corroborated that the Swanson's 1998 nomenclature could apply to the division of mouse hypothalamus, particularly at caudal hypothalamic levels for the identification of the posterior periventricular nucleus (PVp) [41].
